# 1-(2,2-Diphenyl­eth­yl)-1*H*-tetra­zole

**DOI:** 10.1107/S1600536812027675

**Published:** 2012-06-27

**Authors:** Myrvete Tafili-Kryeziu, Kurt Mereiter, Wolfgang Linert, Franz Werner

**Affiliations:** a Vienna University of Technology, Institute of Applied Synthetic Chemistry, Getreidemarkt 9, 1060 Vienna, Austria; b Vienna University of Technology, Institute of Chemical Technologies and Analytics, Getreidemarkt 9, 1060 Vienna, Austria; c Tallinn University of Technology, Department of Chemistry, Akadeemia tee 15, 12618 Tallinn, Estonia

## Abstract

The crystal structure of the title compound, C_15_H_14_N_4_, contains chains of coplanar tetra­zole rings with the chain direction along *b*. These are formed through weak hydrogen bonds, donated by the tetra­zole H atoms and by one of the H atoms of the methyl­ene group, and accepted by two neighbouring N atoms of the adjacent tetra­zole ring. The chains are connected to each other in a staircase-like manner *via* weak hydrogen bonds, donated from the second H atom of the methyl­ene group and accepted by the N atom next to the C atom in the tetra­zole ring. The resulting layers are parallel to the *bc* plane.

## Related literature

For the synthesis, see Kamiya & Saito (1973 ▶). For crystal structure studies of 1*H*-tetra­zol-1-yl compounds, see Absmeier *et al.* (2006 ▶); Grunert *et al.* (2005 ▶); Werner *et al.* (2009 ▶).
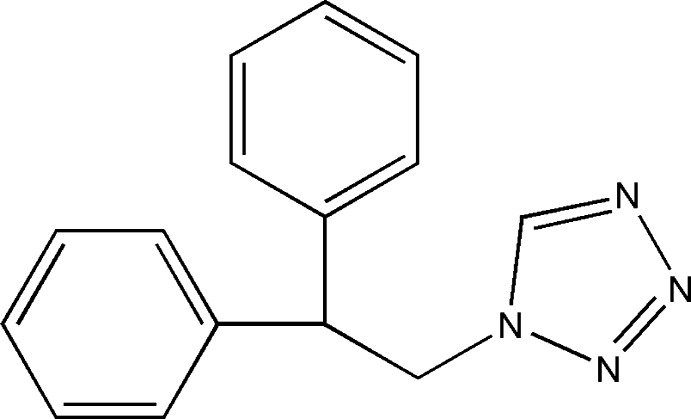


## Experimental

### 

#### Crystal data

C_15_H_14_N_4_
*M* *_r_* = 250.30
Monoclinic, 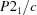

*a* = 12.5289 (6) Å
*b* = 10.4157 (5) Å
*c* = 11.0085 (5) Å
β = 107.906 (1)°
*V* = 1366.99 (11) Å^3^
*Z* = 4
Mo *K*α radiation
μ = 0.08 mm^−1^
*T* = 296 K
0.45 × 0.40 × 0.35 mm

#### Data collection

Bruker APEXII CCD diffractometer
Absorption correction: multi-scan (*SADABS*; Sheldrick, 2008*b* ▶) *T*_min_ = 0.89, *T*_max_ = 0.97
18385 measured reflections
3984 independent reflections
3230 reflections with *I* > 2σ(*I*)
*R*_int_ = 0.019

#### Refinement

*R*[*F* ^2^ > 2σ(*F* ^2^)] = 0.050
*wR*(*F* ^2^) = 0.148
*S* = 1.04
3984 reflections
172 parameters
H-atom parameters constrained
Δρ_max_ = 0.22 e Å^−3^
Δρ_min_ = −0.16 e Å^−3^

### 

Data collection: *APEX2* (Bruker, 2011 ▶); cell refinement: *SAINT* (Bruker, 2009 ▶); data reduction: *SAINT*; program(s) used to solve structure: *SHELXS97* (Sheldrick, 2008*a* ▶); program(s) used to refine structure: *SHELXL97* (Sheldrick, 2008*a* ▶); molecular graphics: *ATOMS* (Dowty, 2006 ▶), *Mercury* (Macrae *et al.*, 2006 ▶) and *VESTA* (Momma & Izumi, 2008 ▶); software used to prepare material for publication: *SHELXL97*.

## Supplementary Material

Crystal structure: contains datablock(s) global, I. DOI: 10.1107/S1600536812027675/fj2563sup1.cif

Structure factors: contains datablock(s) I. DOI: 10.1107/S1600536812027675/fj2563Isup2.hkl

Supplementary material file. DOI: 10.1107/S1600536812027675/fj2563Isup3.cml

Additional supplementary materials:  crystallographic information; 3D view; checkCIF report

## Figures and Tables

**Table 1:** Hydrogen-bond geometry (Å, °)

*D*—H⋯*A*	*D*—H	H⋯*A*	*D*⋯*A*	*D*—H⋯*A*
C1—H1⋯N3^i^	0.93	2.61	3.4690 (19)	153
C2—H2*B*⋯N4^i^	0.97	2.50	3.4622 (17)	174
C2—H2*A*⋯N4^ii^	0.97	2.56	3.5155 (17)	169

Symmetry codes: (i) 
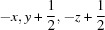
; (ii) 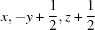.

**Fig. 1.**
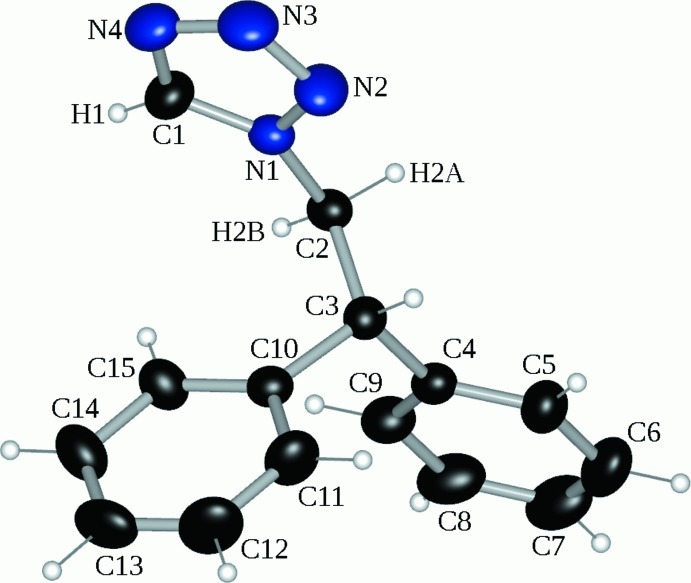
Molecular moiety in the crystal structure of the title compound. Displacement ellipsoids for non–H atoms are drawn at the 33% probability level. Hydrogen atoms involved in weak hydrogen bonding are labelled.

**Fig. 2.**
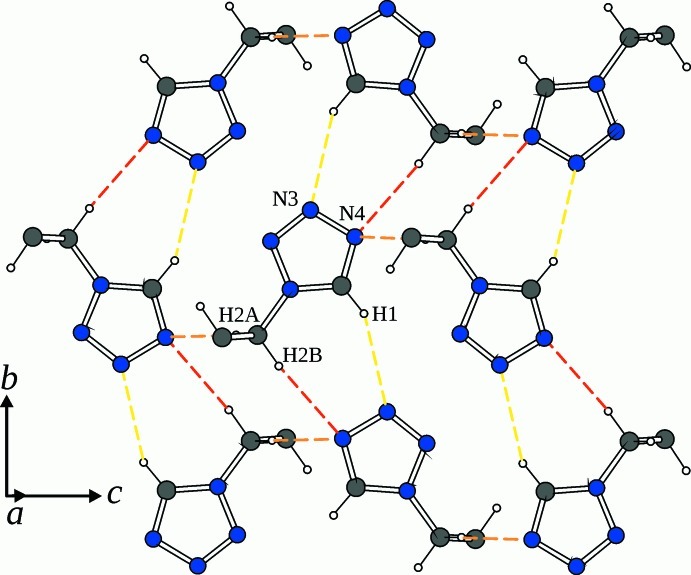
Weak hydrogen bonds in the crystal structure of the title compound, viewed perpedicular to the layers formed by adjacent tetrazolyl rings (yellow C1—H1···N3, red C2—H2B···N4, orange C2—H2A···N4; phenyl rings are omitted for clarity).

**Fig. 3.**
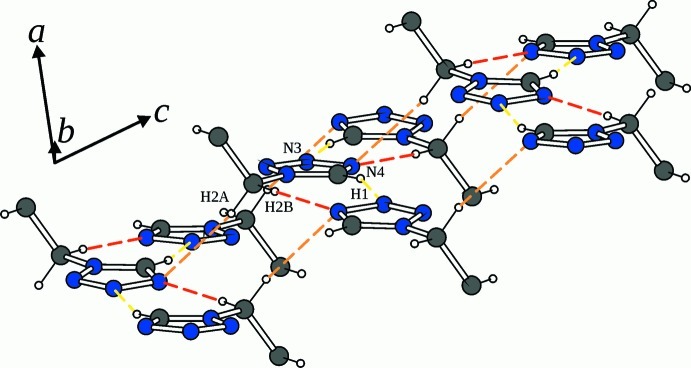
View of the weak hydrogen bonds in the crystal structure of the title compound, showing the staircase-like arrangement (yellow C1—H1···N3, red C2—H2B···N4, orange C2—H2A···N4; phenyl rings are omitted for clarity).

**Fig. 4.**
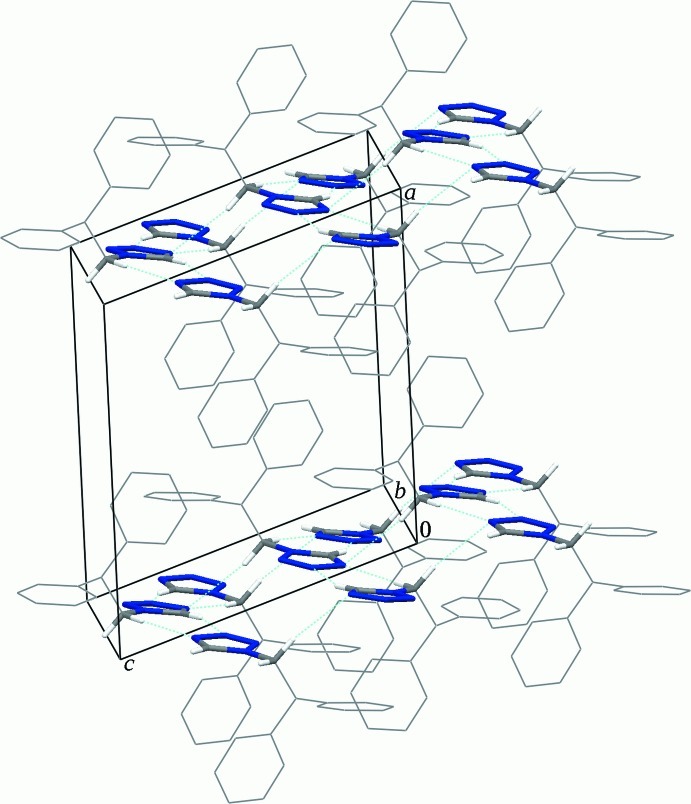
Packing diagram of the title compound. Weak hydrogen bonds are drawn with cyan dashed lines, only those hydrogen atoms involved in weak interactions are shown and the unit cell is outlined.

**Fig. 5.**
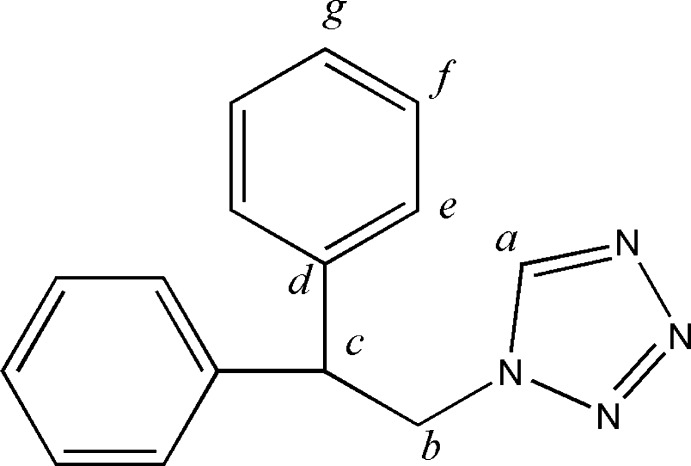
Labelling scheme for the assignment of the NMR chemical shifts.

## supplementary crystallographic information

### Comment

In continuation of the crystallographic characterization of 1*H*–tetrazol–1–yl compounds, intended as potential ligands for Fe(II) spin crossover complexes (Absmeier *et al.*, 2006; Grunert *et al.*, 2005; Werner *et al.*, 2009), the title compound was prepared.

At 296 K the title compound crystallizes in the monoclinic space group *P*2_1_/*c* (No. 14), with one molecule in the asymmetric unit (Fig. 1). Bond lenghts and bond angles in the molecule adopt typical values. The point group symmetry of the free molecule is *C*_s_. Owing to intermolecular interactions this symmetry is lowered to *C*_1_ in the crystalline solid, which can be readily seen from the (+)-synclinal arrangement of the tetrazolyl ring and the phenyl ring C10_···_C15 [N1—C2—C3—C10 = 60.68 (12)°] and the out–of–plane (plane defined by N1, C2 and C3) twist of the tetrazolyl ring [N2—N1—C2—C3 = 63.98 (14)°].

In the crystal the main type of interaction are three sets of weak hydrogen bonds between the tetrazolyl rings (C1—H1···N3) and the tetrazolyl rings and the methylenic H atoms (C2—H2A···N4 and C2—H2B···N4). Through the coplanar interactions C1—H1···N3 and C2—H2B···N4 chains of tetrazolyl rings are formed parallel to the *b*-axis (Fig. 2), whereas C2—H2A···N4 connects these chains in a staircase-like manner (Fig. 3) resulting in the formation of layers parallel to the *bc*-plane with the phenyl rings pointing outwards. The layers are loosely held together by C—H···π interactions (Fig. 4).

### Experimental

The title compound was prepared according to the general procedure given by Kamiya & Saito (1973). All chemicals were used as supplied without further purification. Elemental analyses were performed on a Perkin Elmer 2400 CHN Elemental Analyzer. NMR-spectra were measured in DMSO-d_6_ with a Bruker DPX-200 spectrometer at 200 MHz (^1^H) and 50 MHz (^13^C) respectively. The chemical shifts (see Fig. 5 for the atom assignment) are calibrated to the solvent.

2,2-Diphenylethylamine (4.93 g, 25 mmol, Aldrich, 96%), NaN_3_ (3.25 g, 50 mmol, Fluka, 99%) and triethyl orthoformate (7.42 g, 50 mmol, Acros, 98%) were dissolved in 60 ml of acetic acid (Fluka, 99.8%) and heated to 85–90°C for 24 h. After evaporation of acetic acid under reduced pressure, 70 ml of 2 N hydrochloric acid was added to the residue. The solution was extracted three times with dichloromethane, the combined organic layers were washed with water and saturated aqueous solutions of NaHCO_3_ and NaCl. The organic phase was dried with Na_2_SO_4_, filtered and the solvent was evaporated under reduced pressure. The raw product was recrystallized from methanol. Yield 2.88 g (46%), m.p. 115°C. Single crystals were grown by slow evaporation from a solution of the tetrazole in methanol at room temperature over two days.

Elemental analysis C_15_H_14_N_4_ Calc.: C 71.98, H 5.64, N 22.38. Found: C 72.10, H 5.38, N 21.91%. ^1^H-NMR (DMSO-d_6_) δ [p.p.m.]: 4.73 (*t*, ^3^*J*=8.4 Hz, 1H, H*c*), 5.21 (*d*, ^3^*J*=8.5 Hz, 2H, H*b*), 7.15, 7.19, 7.22, 7.25, 7.29, 7.32, 7.36, 7.40 (*m*, 10H, H*e*–*g*), 9.24 (*s*, 1H, H*a*). ^13^C-NMR (DMSO-d_6_) δ [p.p.m.]: 50.5, 50.8 (C*b*–*c*), 127.0 (C*g*), 127.8 (C*e*), 128.7 (C*f*), 140.7 (C*d*), 144.0 (C*a*).

### Refinement

Hydrogen atoms were included at calculated positions and treated as riding on their base atoms with *d*(C—H)= 0.97 (CH_2_), 0.98 (CH) or 0.93 Å (CH_arom_) and *U*_iso_(H)=1.2*U*_eq_(C). Reflection 020 was omitted because of its large Δ(*F*^2^)/e.s.d. value.

### Crystal data

**Table:**

C_15_H_14_N_4_	*F*(000) = 528
*M**_r_* = 250.30	*D*_x_ = 1.216 Mg m^−^^3^
Monoclinic, *P*2_1_/*c*	Mo *K*α radiation, λ = 0.71073 Å
Hall symbol: -P 2ybc	Cell parameters from 8429 reflections
*a* = 12.5289 (6) Å	θ = 2.6–30.0°
*b* = 10.4157 (5) Å	µ = 0.08 mm^−^^1^
*c* = 11.0085 (5) Å	*T* = 296 K
β = 107.906 (1)°	Block, colourless
*V* = 1366.99 (11) Å^3^	0.45 × 0.40 × 0.35 mm
*Z* = 4	

### Data collection

**Table:**

Bruker APEXII CCD diffractometer	3984 independent reflections
Radiation source: fine-focus sealed tube	3230 reflections with *I* > 2σ(*I*)
Graphite monochromator	*R*_int_ = 0.019
φ and ω scans	θ_max_ = 30.1°, θ_min_ = 3.4°
Absorption correction: multi-scan (*SADABS*; Sheldrick, 2008*b*)	*h* = −17→17
*T*_min_ = 0.89, *T*_max_ = 0.97	*k* = −14→14
18385 measured reflections	*l* = −15→15

### Refinement

**Table:**

Refinement on *F*^2^	Primary atom site location: structure-invariant direct methods
Least-squares matrix: full	Secondary atom site location: difference Fourier map
*R*[*F*^2^ > 2σ(*F*^2^)] = 0.050	Hydrogen site location: inferred from neighbouring sites
*wR*(*F*^2^) = 0.148	H-atom parameters constrained
*S* = 1.04	*w* = 1/[σ^2^(*F*_o_^2^) + (0.0655*P*)^2^ + 0.2149*P*] where *P* = (*F*_o_^2^ + 2*F*_c_^2^)/3
3984 reflections	(Δ/σ)_max_ < 0.001
172 parameters	Δρ_max_ = 0.22 e Å^−^^3^
0 restraints	Δρ_min_ = −0.16 e Å^−^^3^

### Special details

**Table:**

Experimental. Bruker Kappa *APEX2* CCD diffractometer, full-sphere data collection.
Geometry. All e.s.d.'s (except the e.s.d. in the dihedral angle between two l.s. planes) are estimated using the full covariance matrix. The cell e.s.d.'s are taken into account individually in the estimation of e.s.d.'s in distances, angles and torsion angles; correlations between e.s.d.'s in cell parameters are only used when they are defined by crystal symmetry. An approximate (isotropic) treatment of cell e.s.d.'s is used for estimating e.s.d.'s involving l.s. planes.
Refinement. Refinement of *F*^2^ against ALL reflections. The weighted *R*-factor *wR* and goodness of fit *S* are based on *F*^2^, conventional *R*-factors *R* are based on *F*, with *F* set to zero for negative *F*^2^. The threshold expression of *F*^2^ > σ(*F*^2^) is used only for calculating *R*-factors(gt) *etc*. and is not relevant to the choice of reflections for refinement. *R*-factors based on *F*^2^ are statistically about twice as large as those based on *F*, and *R*- factors based on ALL data will be even larger.

### Fractional atomic coordinates and isotropic or equivalent isotropic displacement parameters (Å^2^)

**Table:**

	*x*	*y*	*z*	*U*_iso_*/*U*_eq_	
N1	0.06288 (8)	0.25473 (9)	0.39090 (9)	0.0503 (2)	
N2	0.07582 (12)	0.13614 (12)	0.43964 (12)	0.0755 (4)	
N3	0.03463 (12)	0.05898 (12)	0.34413 (14)	0.0804 (4)	
N4	−0.00552 (11)	0.12529 (13)	0.23470 (12)	0.0724 (3)	
C1	0.01312 (12)	0.24583 (14)	0.26661 (13)	0.0630 (3)	
H1	−0.0057	0.3149	0.2105	0.076*	
C2	0.09677 (9)	0.36869 (11)	0.47103 (11)	0.0493 (2)	
H2A	0.0581	0.3693	0.5351	0.059*	
H2B	0.0740	0.4448	0.4186	0.059*	
C3	0.22336 (9)	0.37415 (10)	0.53767 (10)	0.0464 (2)	
H3	0.2443	0.2965	0.5899	0.056*	
C4	0.24642 (9)	0.48874 (12)	0.62769 (11)	0.0506 (3)	
C5	0.29138 (12)	0.47133 (17)	0.75794 (13)	0.0707 (4)	
H5	0.3081	0.3892	0.7915	0.085*	
C6	0.31156 (15)	0.5784 (2)	0.83910 (16)	0.0925 (6)	
H6	0.3422	0.5670	0.9268	0.111*	
C7	0.28658 (15)	0.6999 (2)	0.7905 (2)	0.0903 (6)	
H7	0.3007	0.7703	0.8451	0.108*	
C8	0.24121 (13)	0.71720 (16)	0.66276 (19)	0.0804 (5)	
H8	0.2235	0.7995	0.6300	0.096*	
C9	0.22120 (11)	0.61269 (12)	0.58099 (14)	0.0617 (3)	
H9	0.1904	0.6257	0.4936	0.074*	
C10	0.29411 (8)	0.37823 (10)	0.44771 (10)	0.0458 (2)	
C11	0.40353 (11)	0.33206 (17)	0.49185 (14)	0.0722 (4)	
H11	0.4298	0.2966	0.5732	0.087*	
C12	0.47312 (12)	0.3379 (2)	0.41749 (19)	0.0919 (6)	
H12	0.5460	0.3068	0.4490	0.110*	
C13	0.43670 (13)	0.38890 (17)	0.29785 (18)	0.0805 (5)	
H13	0.4848	0.3937	0.2483	0.097*	
C14	0.32823 (14)	0.43332 (14)	0.25067 (15)	0.0701 (4)	
H14	0.3024	0.4666	0.1684	0.084*	
C15	0.25730 (11)	0.42836 (12)	0.32603 (12)	0.0563 (3)	
H15	0.1843	0.4592	0.2940	0.068*	

### Atomic displacement parameters (Å^2^)

**Table:**

	*U*^11^	*U*^22^	*U*^33^	*U*^12^	*U*^13^	*U*^23^
N1	0.0485 (5)	0.0525 (5)	0.0533 (5)	−0.0114 (4)	0.0206 (4)	−0.0081 (4)
N2	0.0969 (9)	0.0575 (6)	0.0699 (7)	−0.0239 (6)	0.0225 (6)	−0.0022 (5)
N3	0.0952 (9)	0.0597 (7)	0.0895 (9)	−0.0277 (6)	0.0329 (7)	−0.0172 (6)
N4	0.0727 (7)	0.0758 (8)	0.0703 (7)	−0.0213 (6)	0.0242 (6)	−0.0241 (6)
C1	0.0632 (7)	0.0674 (8)	0.0556 (7)	−0.0093 (6)	0.0141 (5)	−0.0115 (6)
C2	0.0484 (5)	0.0522 (6)	0.0515 (6)	−0.0071 (4)	0.0214 (4)	−0.0102 (4)
C3	0.0499 (5)	0.0438 (5)	0.0453 (5)	−0.0031 (4)	0.0146 (4)	−0.0017 (4)
C4	0.0465 (5)	0.0573 (6)	0.0506 (5)	−0.0084 (4)	0.0189 (4)	−0.0114 (5)
C5	0.0712 (8)	0.0886 (10)	0.0520 (7)	−0.0096 (7)	0.0186 (6)	−0.0095 (7)
C6	0.0856 (11)	0.1339 (18)	0.0576 (8)	−0.0170 (11)	0.0215 (7)	−0.0333 (10)
C7	0.0762 (9)	0.0991 (13)	0.0996 (13)	−0.0139 (9)	0.0326 (9)	−0.0541 (11)
C8	0.0698 (8)	0.0656 (8)	0.1080 (13)	−0.0083 (7)	0.0306 (8)	−0.0332 (8)
C9	0.0611 (7)	0.0550 (7)	0.0701 (8)	−0.0065 (5)	0.0217 (6)	−0.0131 (6)
C10	0.0442 (5)	0.0420 (5)	0.0521 (5)	−0.0029 (4)	0.0164 (4)	−0.0079 (4)
C11	0.0502 (6)	0.0951 (11)	0.0667 (8)	0.0103 (6)	0.0110 (6)	−0.0048 (7)
C12	0.0472 (7)	0.1337 (16)	0.0969 (12)	0.0088 (8)	0.0253 (7)	−0.0167 (11)
C13	0.0695 (8)	0.0884 (11)	0.1028 (12)	−0.0155 (7)	0.0549 (9)	−0.0259 (9)
C14	0.0884 (10)	0.0626 (8)	0.0741 (8)	−0.0027 (7)	0.0470 (7)	0.0009 (6)
C15	0.0588 (6)	0.0527 (6)	0.0641 (7)	0.0081 (5)	0.0285 (5)	0.0063 (5)

### Geometric parameters (Å, º)

**Table:**

N1—C1	1.3209 (16)	C6—H6	0.9300
N1—N2	1.3365 (15)	C7—C8	1.357 (3)
N1—C2	1.4619 (13)	C7—H7	0.9300
N2—N3	1.2978 (17)	C8—C9	1.3854 (18)
N3—N4	1.3449 (19)	C8—H8	0.9300
N4—C1	1.3054 (17)	C9—H9	0.9300
C1—H1	0.9300	C10—C15	1.3783 (17)
C2—C3	1.5301 (15)	C10—C11	1.3918 (16)
C2—H2A	0.9700	C11—C12	1.368 (2)
C2—H2B	0.9700	C11—H11	0.9300
C3—C10	1.5193 (14)	C12—C13	1.362 (3)
C3—C4	1.5212 (15)	C12—H12	0.9300
C3—H3	0.9800	C13—C14	1.377 (2)
C4—C5	1.3818 (18)	C13—H13	0.9300
C4—C9	1.3896 (18)	C14—C15	1.3906 (17)
C5—C6	1.403 (2)	C14—H14	0.9300
C5—H5	0.9300	C15—H15	0.9300
C6—C7	1.372 (3)		
			
C1—N1—N2	108.14 (11)	C5—C6—H6	119.7
C1—N1—C2	129.72 (11)	C8—C7—C6	119.97 (15)
N2—N1—C2	122.09 (10)	C8—C7—H7	120.0
N3—N2—N1	106.15 (12)	C6—C7—H7	120.0
N2—N3—N4	110.72 (12)	C7—C8—C9	120.21 (17)
C1—N4—N3	105.44 (11)	C7—C8—H8	119.9
N4—C1—N1	109.55 (13)	C9—C8—H8	119.9
N4—C1—H1	125.2	C8—C9—C4	120.96 (14)
N1—C1—H1	125.2	C8—C9—H9	119.5
N1—C2—C3	112.67 (9)	C4—C9—H9	119.5
N1—C2—H2A	109.1	C15—C10—C11	118.00 (11)
C3—C2—H2A	109.1	C15—C10—C3	123.79 (10)
N1—C2—H2B	109.1	C11—C10—C3	118.18 (11)
C3—C2—H2B	109.1	C12—C11—C10	121.06 (15)
H2A—C2—H2B	107.8	C12—C11—H11	119.5
C10—C3—C4	111.76 (8)	C10—C11—H11	119.5
C10—C3—C2	114.52 (9)	C13—C12—C11	120.68 (14)
C4—C3—C2	107.62 (9)	C13—C12—H12	119.7
C10—C3—H3	107.6	C11—C12—H12	119.7
C4—C3—H3	107.6	C12—C13—C14	119.61 (13)
C2—C3—H3	107.6	C12—C13—H13	120.2
C5—C4—C9	118.66 (12)	C14—C13—H13	120.2
C5—C4—C3	120.54 (12)	C13—C14—C15	120.00 (14)
C9—C4—C3	120.79 (11)	C13—C14—H14	120.0
C4—C5—C6	119.52 (16)	C15—C14—H14	120.0
C4—C5—H5	120.2	C10—C15—C14	120.64 (12)
C6—C5—H5	120.2	C10—C15—H15	119.7
C7—C6—C5	120.67 (16)	C14—C15—H15	119.7
C7—C6—H6	119.7		
			
C1—N1—N2—N3	0.47 (16)	C5—C6—C7—C8	0.4 (3)
C2—N1—N2—N3	178.18 (11)	C6—C7—C8—C9	−0.7 (2)
N1—N2—N3—N4	−0.49 (16)	C7—C8—C9—C4	0.3 (2)
N2—N3—N4—C1	0.32 (17)	C5—C4—C9—C8	0.42 (19)
N3—N4—C1—N1	−0.01 (16)	C3—C4—C9—C8	179.46 (12)
N2—N1—C1—N4	−0.28 (16)	C4—C3—C10—C15	−95.17 (13)
C2—N1—C1—N4	−177.76 (11)	C2—C3—C10—C15	27.54 (15)
C1—N1—C2—C3	−118.85 (13)	C4—C3—C10—C11	82.93 (13)
N2—N1—C2—C3	63.98 (14)	C2—C3—C10—C11	−154.37 (11)
N1—C2—C3—C10	60.68 (12)	C15—C10—C11—C12	1.0 (2)
N1—C2—C3—C4	−174.40 (9)	C3—C10—C11—C12	−177.25 (15)
C10—C3—C4—C5	−116.60 (12)	C10—C11—C12—C13	−0.2 (3)
C2—C3—C4—C5	116.84 (12)	C11—C12—C13—C14	−0.9 (3)
C10—C3—C4—C9	64.38 (13)	C12—C13—C14—C15	1.3 (2)
C2—C3—C4—C9	−62.18 (13)	C11—C10—C15—C14	−0.55 (19)
C9—C4—C5—C6	−0.7 (2)	C3—C10—C15—C14	177.55 (11)
C3—C4—C5—C6	−179.78 (12)	C13—C14—C15—C10	−0.6 (2)
C4—C5—C6—C7	0.3 (2)		

### Hydrogen-bond geometry (Å, º)

**Table:**

*D*—H···*A*	*D*—H	H···*A*	*D*···*A*	*D*—H···*A*
C1—H1···N3^i^	0.93	2.61	3.4690 (19)	153
C2—H2*B*···N4^i^	0.97	2.50	3.4622 (17)	174
C2—H2*A*···N4^ii^	0.97	2.56	3.5155 (17)	169

Symmetry codes: (i) −*x*, *y*+1/2, −*z*+1/2; (ii) *x*, −*y*+1/2, *z*+1/2.
